# Role of initial system-bath correlation on coherence trapping

**DOI:** 10.1038/srep13359

**Published:** 2015-08-25

**Authors:** Ying-Jie Zhang, Wei Han, Yun-Jie Xia, Yan-Mei Yu, Heng Fan

**Affiliations:** 1 Shandong Provincial Key Laboratory of Laser Polarization and Information Technology, Department of Physics, Qufu Normal University, Qufu 273165, China; 2 Beijing National Laboratory of Condensed Matter Physics, Institute of Physics, Chinese Academy of Sciences, Beijing 100190, China; 3 Innovative Center of Quantum Matter, Beijing 100190, China

## Abstract

We study the coherence trapping of a qubit correlated initially with a non-Markovian bath in a pure dephasing channel. By considering the initial qubit-bath correlation and the bath spectral density, we find that the initial qubit-bath correlation can lead to a more efficient coherence trapping than that of the initially separable qubit-bath state. The stationary coherence in the long time limit can be maximized by optimizing the parameters of the initially correlated qubit-bath state and the bath spectral density. In addition, the effects of this initial correlation on the maximal evolution speed for the qubit trapped to its stationary coherence state are also explored.

Quantum state takes the form of superposition which leads to quantum coherence. Quantum coherence plays a central role in the applications of quantum physics and quantum information science^1^,^2^. However, it is fragile due to interactions of the environment. Understanding of quantum coherence dynamics of an open system is a very important task in many areas of physics ranging from quantum optics to quantum information processing. It is known that many quantum open systems exhibit non-Markovian behavior with a flow of information from the environment back to the system^3^,^4^,^5^,^6^,^7^. This presence of non-Markovian effects can induce the long-lasting coherence in biological surroundings^8^,^9^ and the steady state entanglement in the coherently coupled dimer systems or the thermal equilibrium states^10^,^11^. In this report, we would mainly consider non-Markovian effects on the long-lived coherence of the open system. And then, by considering the pure dephasing non-Markovian bath, decay of quantum coherence of the system would be terminated in a finite time, such that the system can partly retain coherence in the long time limit. This new phenomenon, known as *coherence trapping*^10^,^12^, is important for quantum information processing since the effective long-time quantum coherence of the system is preserved. Coherence trapping of a quantum system is mainly related to the open dynamics, and is generally analyzed in the fact that the system and bath are initially separable. As is well known, however, the initial system-bath correlations are important for the dynamics of the open systems. The distinguishability of quantum states would increase in the presence of initial system-bath correlations^13^,^14^. The information flow between the system and its bath and the corresponding degree of non-Markovianity can also be influenced by the initial correlations^15^,^16^,^17^,^18^. On the other hand, the standard master equation approach to open systems may not be appropriate unless a product state is explicitly prepared^19^,^20^,^21^,^22^,^23^,^24^. Besides, the initial system-bath correlations can also allow for new control channels of open quantum systems with incoherent^25^ or coherent light^26^,^27^. So the coherence trapping of an open system due to the initial system-bath correlations should be studied both physically and methodologically.

In this paper, we will concentrate on the following questions: how do the initial system-bath correlations affect coherence trapping of the system? which form of the initially correlated system-bath state can maximize the stationary coherence of the system? We consider the pure dephasing model of a qubit initially correlated with a zero-temperature Ohmic-Like bath. We will show that the initial qubit-bath correlation can lead to the more efficient coherence trapping, while the lower initial coherence of the qubit is induced by this initial correlation. In the long time limit, the stationary coherence of the qubit can be maximized by choosing the optimal parameters of the initially correlated qubit-bath state and the optimal Ohmicity parameter of the bath.

Furthermore, the task to drive an initial state to a prescribed target state in the shortest possible time is significant for quantum control in many areas of physics, such as quantum computation^28^, fast population transfer in quantum optics^29^, and quantum optimal control protocols^30^,^31^. This minimum evolution time, which is defined as quantum speed limit (QSL) time^32^,^33^,^34^,^35^,^36^,^37^,^38^,^39^,^40^,^41^,^42^,^43^,^44^,^45^, is a key method in characterizing the maximal speed of evolution of quantum systems. Here in order to speed up the evolution from an initial coherence state to its stationary coherence state, we further focus on the interactions of the initial qubit-bath correlated state, the spectral density function of the bath and the QSL time. Remarkably, we find that the initial qubit-bath correlation can reduce the QSL time for the occurrence of coherence trapping. The maximal evolution speed for the qubit trapped to its stationary coherence state can also be controlled by optimizing the parameters of the initial qubit-bath correlated state and the bath spectral density function.

## Results

### Model

Let us consider an exactly solvable model, in which the process of energy dissipation is negligible and only pure depahsing is a mechanism for decoherence of the qubit. The associated Hamiltonian reads (setting *ħ* = 1),


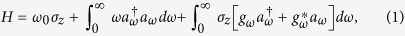


where the operator *σ*_*z*_ is defined by 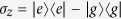, associated with the upper level |*e*〉 and the lower level |*g*〉 of the qubit; *a*_*ω*_ and 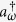 are the bosonic annihilation and creation operators for the bath, which is characterized by the frequency *ω*; *g*_*ω*_ is the coupling constant of the interaction of the qubit with the bath, and 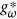 is the complex conjugate to *g*_*ω*_. The Hamiltonian in Eq. (1) can be rewritten in the block-diagonal structure^46^,^47^ *H* = *diag*[*H*_*e*_, *H*_*g*_], where 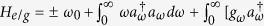 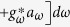.

Here, we consider the situation where a correlated initial state of the qubit-bath system in the form^14^,


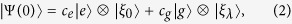


with the non-zero complex numbers *c*_*g*/*e*_ are satisfied |*c*_*e*_|^2^ + |*c*_*g*_|^2^ = 1. Here, in order to introduce the initial coherence of the qubit, we have considered both the ground |*g*〉 and excited |*e*〉 states of the qubit in the above equation. And we assume that |*ξ*_0_〉 is a bath ground state and 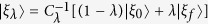 is a bath superposition state of the ground state |*ξ*_0_〉 and a coherent state 
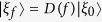
. The displacement operator *D*(*f*) reads 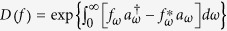 for an arbitrary square-integrable function *f*. The constant 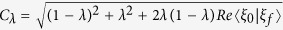 normalizes the state |*ξ*_*λ*_〉, where *R*_*e*_ is a real part of 〈*ξ*_0_|*ξ*_*f*_〉 in the bath Hilbert space. The correlation parameter *λ* ∈ [0, 1] determines the initial correlation of the qubit and bath. Through performing the Hamiltonian described in Eq. (1), the state of the total system at any time *t* is given by 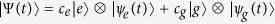, where 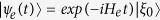 and 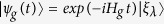. Then the reduced density matrix *ρ*_*λ*_(*t*) of the qubit at time *t* reads, 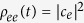, 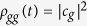 and 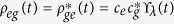, with the dephasing rate ϒ_*λ*_(*t*).

The qubit dynamics is closely dictated by the spectral density function characterising the qubit-bath interaction. In the following the bath can be described by the family of Ohmic-Like spectra 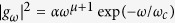, with *ω*_*c*_ being the cutoff frequency and *α* > 0 a dimensionless coupling constant. By changing the *μ*-parameter, one goes from sub-Ohmic baths (−1 < *μ* < 0) to Ohmic (*μ* = 0) and super-Ohmic (*μ* > 0) baths, respectively. And so far, some experimental implementations of the Ohmic-Like baths have been proposed in biological samples for sub-Ohmic baths^48^ and super-Ohmic baths^25^,^49^. Furthermore, the coherent state |*ξ*_*f*_〉 can be calculated by the spectral density function 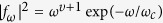, with *υ* > 0. So the initial state of the qubit-bath system can be controlled by the parameters *λ* and *υ*. For the case *λ* = 0 the qubit and the bath are initially uncorrelated, the dephasing rate can be obtained, ϒ_0_(*t*) = exp[−*r*(*t*)]. While for 0 < *λ* ≤ 1 the initial correlation exists in the qubit-bath system, one also finds, 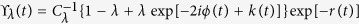, where,


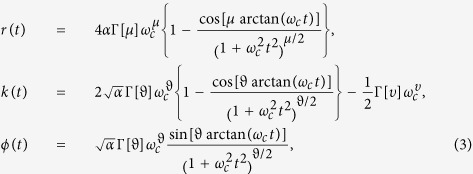


where Γ[·] is the Euler gamma function and the parameter *ϑ* = (*μ* + *υ*)/2.

### Coherence trapping for the qubit

**Figure 1:**
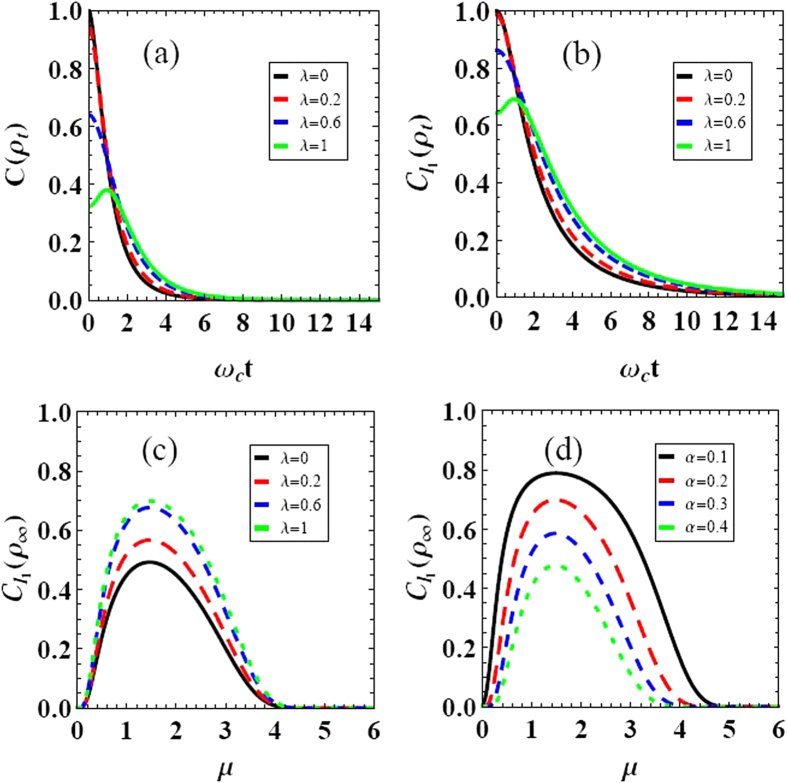
The quantum coherence of the qubit quantified by the relative entropy of coherence *C*(*ρ*) or the intuitive *l*_1_ norm of coherence C_1_1__*ρ* as a function of the bath parameters *α* and *μ*. (**a**) in the sub-Ohmic dephasing bath (*μ* = −0.5) for *C*(*ρ*_*t*_), *α* = 0.2; (**b**) in the sub-Ohmic dephasing bath (*μ* = −0.5) for 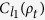, *α* = 0.2; (**c**) for the correlated initial qubit-bath state (*α* = 0.2) for 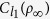; (**d**) for the correlated initial qubit-bath state (*λ* = 1) for 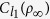. Parameters are chosen as, *υ* = 1.5, and *ω*_*c*_ = 1.

**Figure 2:**
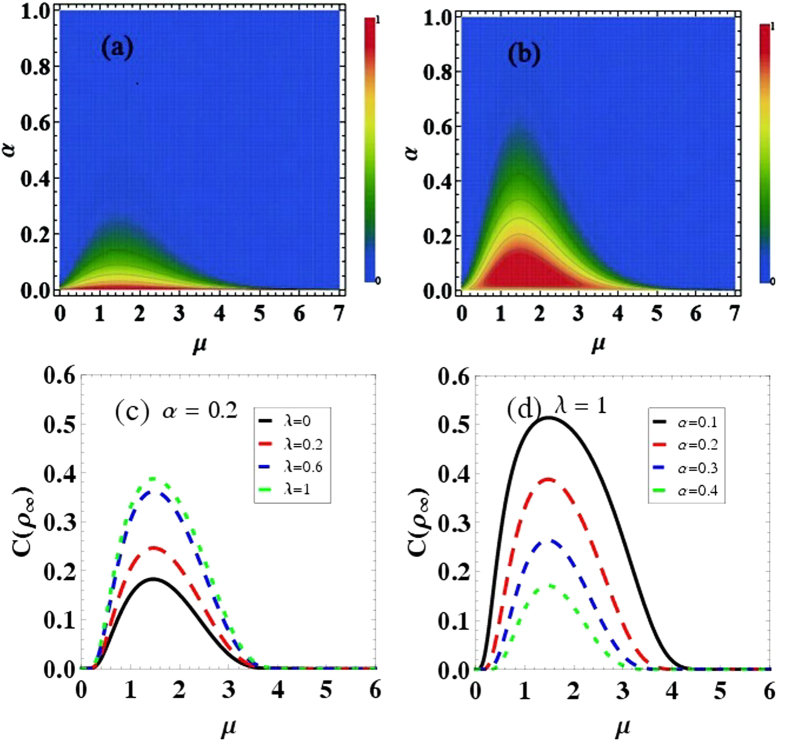
The stationary coherence of the qubit quantified by the relative entropy of coherence *C*(*ρ*_∞_) as a function of the bath parameters *α* and *μ*. (**a**) for the uncorrelated initial qubit-bath state (*λ* = 0); (**b**) for the correlated initial qubit-bath state (*λ* = 1); (**c**) for the correlated initial qubit-bath state (*α* = 0.2); (**d**) for the correlated initial qubit-bath state (*λ* = 1). Parameters are chosen as, *υ* = 1.5, and *ω*_*c*_ = 1.

How to quantify quantum coherence of a quantum system now becomes paramountly important. In recent years, a wide variety of measures of coherence have been proposed^50^,^51^,^52^. Currently, Baumgratz, Cramer and Plenio find that the relative entropy of coherence^50^,


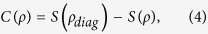


where *S*(*ρ*) is the von Neumann entropy and *ρ*_*diag*_ denotes the state obtained from *ρ* by deleting all off-diagonal elements, and the intuitive *l*_1_ norm of coherence,


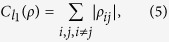


are both general and proper measures of coherence. The relative entropy of coherence *C*(*ρ*) and the intuitive *l*_1_ norm of coherence 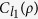 have been chosen to measure the quantum coherence of the reduced density matrix of the qubit in the presence of qubit-bath initial correlation in Figs 1 and 2. By comparing Fig. 1 and 2, it is clear to find that these two measures can have a similar trend for coherence of the qubit by considering the same parameters of the pure dephasing model. So the analysis of coherence trapping in this report would not depend on the choice of coherence measure, and in the following we mainly utilize the relative entropy of coherence *C*(*ρ*) to measure quantum coherence of the qubit.

If there is no correlation in the initial qubit-bath state, the qubit dephasing ϒ_0_(*t*) is characterized by exponential decay of the qubit coherence, hence will predict vanishing coherence in the long time limit in the Ohmic and sub-Ohmic dephasing baths^12^. And even if there exist finite qubit-bath correlations in the initial state, the qubit coherence can also be gradually reduced to zero in the Ohmic and sub-Ohmic dephasing baths, as shown in Fig. 1(a,b). While for the super-Ohmic baths, the qubit dephasing will stop after a finite time, therefore lead to coherence trapping. This behavior can realize the effective long-time coherence protection. In the following, we would mainly see the effect of the initially correlated qubit-bath state on coherence trapping of a qubit in the super-Ohmic bath model. The preparation of this initially correlated qubit-bath state can be obtained by non-local operations with two steps^14^. But these two steps would essentially require sophisticated quantum engineering and precise technics in experiment. And the initial correlations of the qubit-bath system can be controlled by the parameters *c*_*g*/*e*_, *λ* and the function *f*_*ω*_.

We shall examine the decoherence process where the initially correlated qubit-bath state is in the form of Eq. (2), with 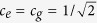. Then the initial coherence of the qubit can be evaluated 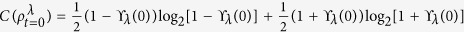, with 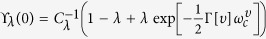. At time *t* = 0, in the case *λ* = 0 the dephasing rate ϒ_0_(0) = 1, while for the correlated initial state we can obtain 0 < ϒ_*λ*_(0) < 1. From the above expression of 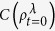, we find that this initial coherence of the qubit is mainly dependent of the parameters *λ* and *v*. We can find that 
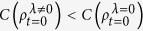
, and the initial correlation of the qubit-bath system can lead to lower initial coherence of the qubit. Furthermore, according to Eq. (3), we also specifically point out that, when the qubit-bath coupling strength *α* tends to zero, the dephasing rates ϒ_0_(*t*) = ϒ_0_(0) and ϒ_*λ*_(*t*) = ϒ_*λ*_(0) are independent of the time parameter *t* and the Ohmicity parameter *μ*. So the initial qubit coherence would not decay in this limit.

On the other hand, to clear the effect of the qubit-bath initial correlation explicitly, we also perform the calculation for the stationary value of coherence trapping in the long time limit. In Fig. 2, we show the stationary coherence *C*(*ρ*_∞_) between the initially uncorrelated *λ* = 0 and correlated *λ* = 1 states as a function of the bath parameters *α* and *μ*. By comparing Fig. 2(a,b), it is clear that the presence of the qubit-bath correlation in the initial state enlarges the region for occurrence of coherence trapping. Moreover, by giving the other parameters, Fig. 2(c) clearly shows that the larger correlation parameter *λ* leads to a more efficient coherence trapping. That is to say, the stationary coherence is higher than that obtained from the initially uncorrelated qubit-bath state. Although the lower initial coherence of the qubit can be induced by the correlation parameter *λ*, the coherences of the bath subsystem and the qubit-bath composite system would appear in the initial qubit-bath state correspondingly. And the larger initial qubit-bath correlation (the larger *λ*) can lead to the stronger non-Markovian dynamics. Then the initial coherence of the bath subsystem would be more transferred to the qubit coherence in the stronger non-Markovian dynamics. That is the main physical reason of the more efficient coherence trapping of the qubit induced by the correlated initial qubit-bath state. Additionally, from Fig. 2 we also can easily find that, the stronger coupling *α* of the qubit to bath diminishes the stationary coherence in the long time limit. And there exists an optimal value of the Ohmicity parameter 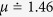 maximizing the stationary coherence in zero temperature bath, which is independent of the coupling constant *α* and the correlation parameter *λ*, as shown in Fig. 2(c,d).

**Figure 3:**
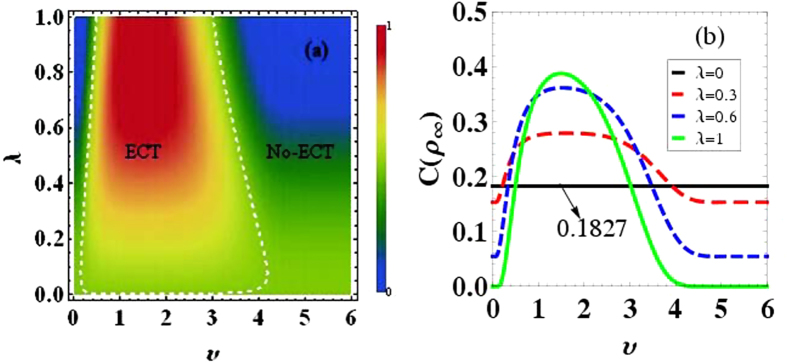
The stationary coherence of the qubit quantified by the relative entropy of coherence *C*(*ρ*_∞_) as a function of the parameters for the initial qubit-bath state *λ* and *υ*. The dashed-white line in (*a*) means *C*(*ρ*_∞_) = 0.1827, which is the dividing line between two regions. Parameters are chosen as, *α* = 0.2, *μ* = 1.46, and *ω*_*c*_ = 1.

Next, by choosing the optimal value *μ* = 1.46 of the super-Ohmic bath, the influence of the parameters characterizing the initially correlated state on coherence trapping is depicted in Fig. 3(a). Two regions, the enhancing of coherence tapping (ECT) (i.e. 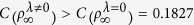) and the no-enhancing of coherence trapping (No-ECT) (i.e. 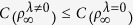), are acquired in the corresponding parameter planes. The dashed-white line 
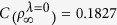
 is the dividing line between these two regions. That is to say, not all but specific initial states |*ξ*_*λ*_〉 can lead to the enhancing coherence trapping. The range of *υ* to gain the enhancing of coherence trapping, would reduce as the correlation parameter *λ* increasing, as shown in Fig. 3(b). So we conclude that, in order to achieve the most efficient coherence in the long time limit, both the optimal Ohmicity parameter *μ* and the optimal state |*ξ*_*λ*_〉 must be satisfied.

### Quantum evolution speed

Since the decay of quantum coherence of the qubit would be terminated in a finite time *t*_*c*_, the qubit would occur coherence trapping when the evolutionary time *t*_*a*_ > *t*_*c*_ in the super-Ohmic bath. Then one may naturally concern the evolution speed between the initial state *ρ*_*λ*_(0) and the stationary coherence state *ρ*_*λ*_(*t*_*c*_). The quantum speed of evolution from *ρ*_*λ*_(0) to its target state *ρ*_*λ*_(*t*_*c*_) can be characterized by QSL time^43^,^44^. The definition of QSL time between an arbitrary initially mixed state *ρ*_0_ and its target state *ρ*_*τ*_ with the actual time *τ*, governed by the master equation 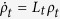, with *L*_*t*_ the positive generator of the dynamical semigroup, is as follows^44^ 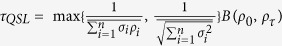, here 0 < *τ*_*QSL*_ < *τ*, and 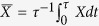, 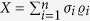, 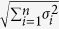, 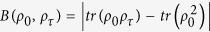 denotes a metric on the space of the initial state *ρ*_0_ and the target state *ρ*_*τ*_ via the so-called relative purity, and *σ*_*i*_ are the singular values of 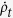 and 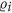 those of the initial mixed state *ρ*_0_. The above expression of *τ*_*QSL*_ can effectually define the minimal evolution time for arbitrary initial states, and also be used to assess quantum evolution speed of open quantum system.

**Figure 4:**
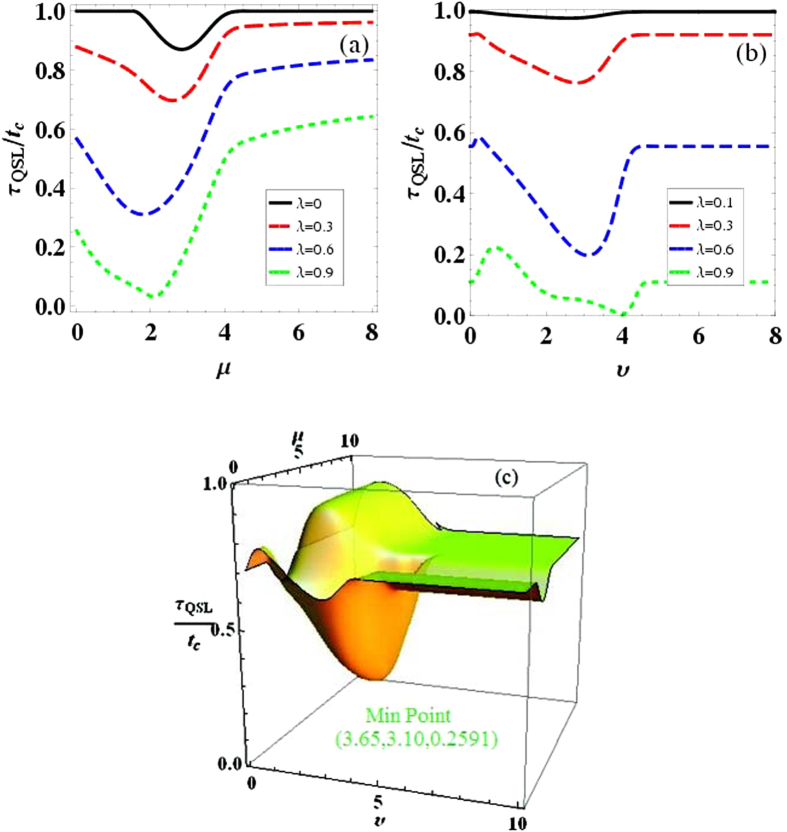
The quantum speed for the evolution from the initial state *ρ*_*λ*_(0) to the stationary coherence state *ρ*_*λ*_(*t*_*c*_), quantified by *τ*_*QSL*_/*t*_*c*_ as a function of the parameters *μ* and *υ*, here the actual trapped time *t*_*a*_ = 3 is independent of the model parameters, and *ω*_*c*_ = 1. Parameters are chosen as, (**a**) *α* = 0.2, *υ* = 2; (**b**) *α* = 0.2, *μ* = 1.46; (**c**) *α* = 0.2, *λ* = 0.5.

Here, we also consider the weights 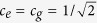 in the initially correlated qubit-bath state in Eq. (2). Then the QSL time for the qubit initial state *ρ*_*λ*_(0) to the trapped stationary coherence state *ρ*_*λ*_(*t*_*c*_) with the actual time *t*_*c*_, can be calculated 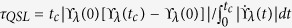, with 0 < *τ*_*QSL*_ < *t*_*c*_. As we all known, the coherence trapping time *t*_*c*_ depends on the model parameters, then the QSL time *τ*_*QSL*_ cannot be easily calculated. Here, we calculate the value of *τ*_*QSL*_/*t*_*c*_ to assess quantum evolution speed of the coherence trapping process. The smaller value of *τ*_*QSL*_/*t*_*c*_ is, the faster quantum speed of the evolution from the qubit initial state to the trapped stationary coherence state is. In order to calculate *τ*_*QSL*_/*t*_*c*_, we choose a finite trapped time *t*_*a*_ > *t*_*c*_, which is independent of the model parameters. When the qubit has been trapped on a stationary coherence state, there exist 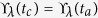 and 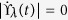 in 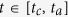. Then we can acquire 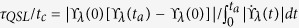. In Fig. 4(a,b), we demonstrate how the QSL time for evolution from *ρ*_*λ*_(0) to *ρ*_*λ*_(*t*_*c*_) can depend on the parameters *μ* and *υ*, with different selected correlation parameter *λ*. Firstly, it is clear that the initial qubit-bath correlation can reduce the QSL time as the value of *λ* increasing. That is to say, the evolution from the initial coherence state to the stationary coherence state, can be speeded up by the initial correlation in the qubit-bath state. And then, another remarkable feature can be acquired: There exist the optimal Ohmicity parameter *μ* or the parameter *υ* of |*ξ*_*f*_〉, which can induce the minimum value of *τ*_*QSL*_/*t*_*c*_. And the optimal parameters *μ* or *υ* are dependent of the correlation parameter *λ*. In Fig. 4(a), when *υ* = 2, the optimal Ohmicity parameter 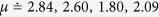 for *λ* = 0, 0.3, 0.6, 0.9, respectively. By choosing *μ* = 1.46, as shown in Fig. 4(b), the optimal parameter for the initial bath state |*ξ*_*f*_〉 can be obtained 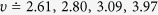 for *λ* = 0.1, 0.3, 0.6, 0.9, respectively.

Furthermore, since both the Ohmicity parameter *μ* and the parameter *υ* of |*ξ*_*f*_〉 can bring about the minimum *τ*_*QSL*_/*t*_*c*_, in the following we would seek the optimal condition (*υ*, *μ*) on the maximal evolution speed of the qubit. Figure 4(c) shows QSL time for *ρ*_*λ*_(0) to *ρ*_*λ*_(*t*_*c*_) as a function of *μ* and *υ*. By a given correlation parameter *λ* = 0.5, we observe that, the minimum *τ*_*QSL*_/*t*_*c*_ can only appear in the region (2 < *υ* < 5, 2 < *μ* < 4). And the optimal values (*υ* = 3.65, *μ* = 3.10) which lead to the minimum value 
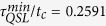
, can be found by accurate numerical calculation. This can be understood that, in order to speed up the evolution speed of the qubit, the Ohmicity parameter *μ* and the parameter *υ* of |*ξ*_*f*_〉 should be optimized. Combined with the above section about coherence trapping, the aim to make the qubit trap in a higher stationary coherence state with the maximal evolution speed, can be attained by choosing the optimal parameters of the initial qubit-bath state (*λ*, *υ*) and the bath spectral density function (*μ*).

## Discussion

In summary, we studied intriguing features of coherence trapping of a qubit with a zero-temperature structured bath by considering the initial qubit-bath correlation. The initial qubit-bath correlation not only leads to a more efficient coherence trapping, but also speeds up the evolution for the occurrence of coherence trapping. Moreover, both the maximum stationary coherence in the long time limit and the minimum QSL time from the initial state to the stationary coherence state, can be acquired by optimizing the parameters of the initially correlated qubit-bath state and the bath spectral density. This physical mechanism leading quickly to a higher stationary coherence would play an important role for implementing quantum simulators^53^ and quantum information processors^54^. Additionally, non-Markovian effects of the bath are the main reason for the results in this report. Recently, by considering the driven dissipative systems^55^, non-Markovian dynamics induced by the time-dependent external fields, can support the generation of out-of-equilibrium steady state entanglement at higher temperatures, larger coupling-to-the-environment constants and lower pumping rates. So it is interesting to consider the relationship between the long-time stationary coherence and non-Markovian dynamics in the driven dissipative systems. However, it is necessary to mention that non-Markovian effects are not the only mechanism for this long-lived coherence in photosynthetic light-harvesting systems^56^,^57^. Three physical features: the small energy gap between excitonic states, the small ratio of the energy gap to the coupling between excitonic states and the effective low-temperature regime, found to be responsible for the long-live coherence in such systems, should also be considered. Finally, it is also worth pointing out that the non-Markovian effects may not monotonically cause the acceleration of the system evolution in the super-Ohmic bath, as shown in Fig. 4(a). This is clearly different from the main result in the damped Jaynes-Cummings model^43^, which shows that the evolution speed can be monotonically increased by non-Markovian effects. So the specific interplay between the evolution speed of the system and the bath non-Markovian effects should be studied under different circumstances. Experimentally, the coherence trapping can be demonstrated by qubit-bath systems like optics^17^, trapped ions^58^ and superconducting qubit^54^,^59^,^60^. And in order to acquire the maximum stationary coherence and the minimum QSL time, there also exist some methods to manipulate the Ohmicity parameter and the initial coherent state in experiment, such as crystals engineered 1D photonic-band-gap micro-cavities^61^ and the superconducting circuit^62^.

## Additional Information

**How to cite this article**: Zhang, Y.-J. *et al.* Role of initial system-bath correlation on coherence trapping. *Sci. Rep.* **5**, 13359; doi: 10.1038/srep13359 (2015).
